# How does emotional exhaustion among Chinese college students affect mental health? A mixed-methods study in Zhejiang, China

**DOI:** 10.3389/fpubh.2025.1669092

**Published:** 2025-10-28

**Authors:** Longyuan Jiang, Lefei Fang, Yuanqi Xu, Qinyu Zhang, Shujing Dai, Jiakun Tian, Wei Wu, Yuan Fang, Meili Zhang, Haiyan Yu

**Affiliations:** School of Medical Humanities and Management, Wenzhou Medical University, Wenzhou, China

**Keywords:** social ecological theory, emotional exhaustion, college students, mental health, SEM, fsQCA

## Abstract

**Introduction:**

This study is based on the social ecology theory and clarifies how emotional exhaustion acts as a proximal mechanism to transmit macro cultural norms, meso institutions and need systems, and micro cognition and behavioral processes to the mental health of college students.

**Methods:**

We conducted a questionnaire survey in 26 universities in Zhejiang Province and received valid responses from 600 students. We used the covariance structural equation model to estimate direct effects and mediating effects, and used fuzzy set qualitative comparative analysis to identify asymmetric configurations sufficient to lead to high-risk outcomes.

**Results:**

The structural equation model showed that the culture of internal competition, academic pressure, employment pressure, rumination thinking, and negative personal behaviors significantly increased emotional exhaustion, while the laid-back culture and relationship needs were not significant. Emotional exhaustion significantly predicted poorer mental health and played a mediating role between each predictor and the outcome. The fuzzy set qualitative comparative analysis identified three types of paths.

**Discussion:**

The results collectively indicate that emotional exhaustion is a transmission hub in the social ecosystem. The evidence of symmetric mean effects and asymmetric configurations mutually corroborates, suggesting the necessity of multi-level intervention and hierarchical implementation.

## Introduction

In the context of heated global competition in higher education and intertwined social uncertainties in the post-epidemic era, the mental health level of college students has become a critical issue affecting individual development and social sustainability (1). However, according to the World Health Organization’s mental health survey of college students in 21 countries (including China), a high prevalence of psychological barriers among college students, with problems such as anxiety and depression being especially prominent (2). The mental health problem of college students has transcended the individual psychological domains to reflect deeper structural contradictions in society and culture, posing a systemic public health challenge (3). Recent meta-analytic data indicate elevated student burnout, with a pooled prevalence of high emotional exhaustion around 56% during and after the pandemic (4) and campus studies in China report that lying flat behaviors are common among undergraduates, while an academic involution atmosphere is associated with stronger stress responses and poorer well-being (5).

Emotional exhaustion refers to feelings of emotional over-extension and depletion and is the core affective component of burnout, yet it is not equivalent to the broader academic-burnout syndrome that also includes cynicism and reduced efficacy (6). In the university context, involution (nei juan) denotes escalating effort under perceived zero-sum competition with diminishing returns (7). Recent Rasch and factor-analytic work provides psychometric support for an Academic Involution Scale among Chinese college students (8). Lying flat (tang ping) describes an attitudinal withdrawal from status competition; a six-item Lying flat Tendency Scale has been developed and validated in Chinese youth samples (8), which strengthens the theoretical footing and enables empirical modeling beyond popular discourse.

At the macro level, recent work has operationalized involution and lying flat with validated instruments and linked these cultural frames to student mental-health correlates. The Academic Involution Scale for College Students shows solid psychometric properties, and the six-item Lying flat Tendency Scale has been validated in youth samples; studies also relate lying flat orientations to well-being indicators, suggesting these are measurable cultural pressures rather than mere rhetoric. At the meso level, perceived campus mental-health climate, workload norms, and competitive cues are prospectively associated with lower psychological distress and higher help-seeking, and meta-analytic evidence shows that university sense of belonging is robustly related to student well-being and academic outcomes, underscoring organizational influences on health. At the micro level, research has focused on individual stressors and behaviors: surges in academic workload coupled with declining performance trigger anxiety and depression (9); social-media dependence exacerbates psychological risk via emotional exhaustion (10) and reduced physical activity limits offline engagement and aggravates mental-health problems (11). Diverse designs—including cross-sectional surveys and large databases (12), longitudinal analyses across time or gender (13), natural experiments, and recent systematic reviews and meta-analyses (14)—have advanced but also compartmentalized the evidence base.

However, prior studies still face several limitations. First, the cross-level integration of macro-cultural frames such as involution (15) and lying flat (16) with meso climates and micro processes remains comparatively underdeveloped, even as psychometric tools for these macro constructs have matured (17). Second, cumulative and nonlinear dynamics are often implied, yet the pathway through which stressors translate into mental health outcomes via emotional resource depletion is not modeled consistently together with macro-level inputs (18). Third, traditional structural equation modeling is well suited to estimating average net effects and indirect paths, but it is not designed to detect asymmetric, configuration-dependent patterns across levels, which limits the detection of equifinality and conjunctural causation (19). These gaps motivate a design that combines cross-level theory, a clear mediator, and complementary analytic tools.

To address these issues, we adopt a socio-ecological perspective because it explains student mental health as the product of nested systems operating in everyday campus life. The approach attends to macrosystem norms, meso settings such as organizational climate and policies, and micro processes including appraisals and regulation, and it emphasizes their reciprocal influences (20). This lens suits our question by tracing cross-level cascades from context to individual experience and by locating both risks and protections across levels (21). Within this framing, emotional exhaustion can be positioned as a proximal mechanism that carries contextual demands into mental-health outcomes, consistent with Job Demands–Resources theory (22). Methodologically, we combine traditional Structural Equation Modeling (SEM) to estimate symmetric directional paths and mediating effects with fuzzy-set Qualitative Comparative Analysis (fsQCA) to identify asymmetric, configuration-dependent sufficient conditions, thereby capturing equifinality and conjunctural causation beyond net-effect models. Accordingly, this study aims to construct a social ecological multilevel model that integrates macro cultural factors (involution culture, lying flat culture), meso organizational factors (survival needs, relational needs, academic pressure, employment pressure), and micro individual factors (rumination, personal behavior) to examine the mediating role of emotional exhaustion, and use SEM to accurately quantify the symmetric paths and mediating effects of macro-meso-micro variables on emotional exhaustion and mental health, and use fsQCA to identify asymmetric configurations under different combinations, revealing multiple high-risk paths. This “symmetrical + asymmetrical” mixed-method strategy not only overcomes the limitations of single-method approaches but also offers a fresh perspective for applying social-ecological theory in educational psychology in theory, thereby providing a robust empirical foundation for designing multi-layered, context-sensitive intervention programs in practice.

## Proposed model and development of the hypotheses

### Theoretical background of emotional exhaustion

Emotional exhaustion refers to a state of profound fatigue resulting from excessive depletion of emotional resources when an individual copes with prolonged work or study stress (23). It is characterized by drained emotional energy, loss of vigor, and a lack of enthusiasm and commitment toward work or academic tasks.

Research on emotional exhaustion has evolved through four stages. In the 1980s, studies focused on medical and service professions (24). Maslach’s seminal work defined occupational burnout as a three-dimensional construct of emotional exhaustion, depersonalization, and reduced personal efficacy, viewing it as a response to chronic workplace stress. In the early 21st century, researchers began to pay attention to emotional exhaustion in educational situations, correlating it with students’ academic stress and role overload (25). After 2010, studies adopted interdisciplinary perspectives, integrating psychology, sociology, and organizational behavior to understand exhaustion. The latest research since 2020 emphasizes the impact of increasingly digital learning environments and the COVID-19 pandemic context, revealing the significantly increased vulnerability of students (20).

Current research examines multiple triggers of emotional exhaustion. Studies have identified factors such as perfectionism (26) and financial stress (27) as important contributors to college student exhaustion. Longitudinal evidence further shows a bidirectional relationship with mental health: emotional exhaustion can predict depression and anxiety, and existing psychological problems can in turn exacerbate exhaustion risk (28). Quantitative research has deepened the understanding of cultural situations—for example, in collectivist cultures like China, high societal expectations for academic achievement amplify emotional exhaustion.

Although the existing literature has provided important perspectives for understanding emotional depletion, some limitations remain. First, most studies have concentrated on specific occupational groups (29), with relatively less focus on college students. Second, interventions have predominantly targeted the individual level, with insufficient attention to the influence of the macro socio-cultural environment. Finally, cross-cultural differences in emotional exhaustion have not been fully explored; scales developed in Western contexts may not adequately capture the effects of academic stress under Confucian cultural backgrounds (30). Therefore, it is a useful supplement to explore the internal mechanism of emotional exhaustion of Chinese college students that potentially affects students’ mental health and learning efficiency and even leads to the symptoms of depression and anxiety, which is a useful supplement to the current academic field.

### Proposed model

We develop a multi-level analytical framework based on Bronfenbrenner’s social ecological theory. This theory emphasizes the multi-level interactions between society and the environment (31), especially focusing on the dynamic interactions between behavior and the environment (32). The framework divides influencing factors into three subsystems: the macro level, including structural context such as cultural norms and institutional policies; the meso level, focusing on group interactions and organizational support systems; and the micro level, referring to individual cognitive and emotional regulation processes (31). Macrocultural factors influence micro-individual behavior by shaping the meso-organizational environment. This multi-dimensional explanatory approach has been widely validated in health behavior research, providing a methodological foundation for analyzing mental health issues within complex social-ecological systems (21).

#### Macro-level

Under Social Norms Theory sociocultural operate on individual behavior through collective norms, including both mainstream behavioral codes and rebellions against the mainstream. Involution culture refers to the unnecessary vicious competition among individuals and the resulting friction as a social phenomenon (33), characterized by individuals continuously exerting effort in a fiercely competitive environment without proportional rewards. It then leads to emotion-regulation failure and emotional exhaustion (34). In Chinese college samples, the Academic Involution Scale shows unidimensional structure and acceptable Rasch and reliability evidence, which supports its operational definition and construct validity in this context (7). On the other hand, lying flat cultures refers to a social phenomenon where individuals work in the minimalistic manner (35). It cope with stress through a low desire or low commitment lifestyle. A six item Lying Flat Tendency Scale has been validated in Chinese youth, indicating reliability and construct validity (16). These frames are culturally specific to contemporary China, yet they map onto general processes of competitive pressure and withdrawal that can appear in other high stakes settings, which suggests potential generalizability across contexts. Importantly, the impact of lying flat is context dependent. Short term disengagement may relieve perceived demand, whereas sustained withdrawal may reduce belonging and social resources and can elevate the risk of emotional exhaustion over time (34). Based on this reasoning, the following hypothesis is proposed:

*H1*: Involution culture has a positive effect on emotional exhaustion.

*H2*: Lying flat culture has a positive effect on emotional exhaustion.

#### Meso-level

Based on the ERG demand theory of Alderfer (36), this study analyzes how the imbalance of the three demands—survival, relationship and growth—aggravates emotional exhaustion from the meso-level. When survival needs are unmet (e.g., economic resource scarcity), persistent anxiety ensues, causing individuals to over-allocate their limited cognitive resources to basic survival concerns (37). This weakens individual’s capacity to cope with other stressors and ultimately leads to emotional exhaustion (38). Unmet relationship needs (for instance, lack of social support) reduce individual stress responses to regulatory thresholds, leading to the accumulation and intensification of negative emotions (39). Additionally, relational conflicts (with teachers, family, etc.) further drain psychological resources, triggering exhaustion (40). In terms of growth needs, academic pressure, and employment pressure are primary sources of developmental anxiety. Academic pressure arises from a disparity between academic expectations and an individual’s time or ability resources, leading to the deprivation of the sense of self-control (41) and forming a vicious circle where efforts fail to produce the desired efficacy (42). Employment pressure induces cognitive overload through career uncertainty (43), causing a dilemma of unclear professional identity, skill adaptation anxiety, and shrinking opportunity structures. These pressures not only directly deplete emotional resources but also foster a pessimistic outlook on the future and weaken psychological resilience, thereby exacerbating emotional exhaustion. Based on this reasoning, the following hypothesis is proposed:

*H3*: Unmet survival needs have a positive effect on emotional exhaustion.

*H4*: Unmet relationship needs have a positive effect on emotional exhaustion.

*H5*: Academic pressure has a positive effect on emotional exhaustion.

*H6*: Employment pressure has a positive effect on emotional exhaustion.

#### Micro-level

Based on the emotion regulation theory of Gross (44), we deconstruct the occurrence mechanism of individual emotional depletion from the two stages of the emotion regulation process. As the failure mode of the regulatory initiation phase, rumination reduces the cognitive focus, amplifies the emotional response, and enables individuals to continuously focus on negative events, leading to the mismatch of attentional resources and repeatedly activating negative emotional memory, thus exacerbating emotional exhaustion (45), forming a self-reinforcing emotional vortex. Non-adaptive individual behavior reflects the failure of the regulatory strategy selection stage, and individuals try to cope with short-term emotional stress, such as smoking, drinking alcohol and staying up late (46). However, such behaviors disrupt physiological function (47), increase long-term psychological resource depletion, and weaken the individual’s ability to adapt to stress (48), ultimately leading to emotional exhaustion (10). Based on this reasoning, the following hypothesis is proposed:

*H7*: Ruminative thinking has a positive effect on emotional exhaustion.

*H8*: Maladaptive personal behavior has a positive effect on emotional exhaustion.

Emotional exhaustion influences mental health in two ways. First, it operates as a proximal strain mechanism that carries contextual demands into health outcomes, consistent with Job Demands and Resources theory, Conservation of Resources theory, and the burnout tradition (22, 37, 49). Second, it has a direct effect on mental health through impaired regulation and heightened anxiety and depressive symptoms, with evidence in student and worker populations (25). Based on this reasoning, the following hypothesis is proposed:

*H9*: Emotional exhaustion mediates the effects of each of the eight aforementioned factors on mental health.

*H10*: Emotional exhaustion has a positive effect on mental health problems.

**Figure 1:**
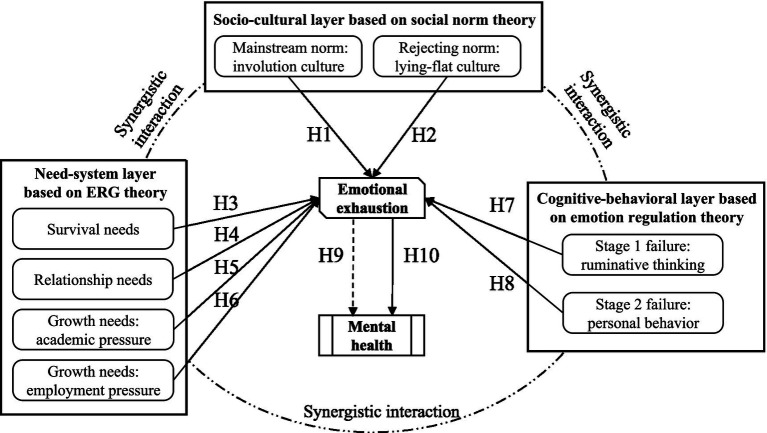
The proposed model. Solid lines indicate direct paths; dashed lines indicate paths via the mediator.

The theoretical framework diagram formed by this study is shown in Figure 1. Macro level cultural frames can permeate the meso layer by shaping the organizational climate around survival and relational needs as well as academic and employment pressures, which in turn condition how micro level processes such as rumination and personal behavior link to emotional exhaustion and mental health. The meso layer can buffer or amplify macro influences through belonging and resource availability, and improvements in organizational climate are longitudinally associated with lower distress and stronger help seeking. At the same time, aggregated micro behaviors and sentiments can feed back to the meso layer and gradually normalize or resist macro prescriptions.

## Methodology

### Sampling and data collection

We selected Zhejiang Province in eastern China as the region for our empirical study. Zhejiang is a representative context due to three socio-ecological characteristics. First, as an economically developed province in eastern China, Zhejiang features a highly competitive market environment and a multi-tiered higher education system (including “Double First-Class” universities, provincial key universities, and numerous vocational colleges). It provides a typical situation that is common in the rapid modernization process of China for students to be exposed to academic competition (“involution”) and employment anxiety (5). Second, Zhejiang exhibits a pronounced developmental gradient: major cities like Hangzhou and Ningbo contrast with less-developed areas such as Lishui and Quzhou. This leads to significant differences in college students’ realistic conditions such as economic status. This phenomenon of uneven regional development is prevalent across all provinces in China, providing an ideal and nationally valuable reference sample for studying the impact of latent variables on mental health (50). Moreover, as the pilot demonstration area of the construction of the national social psychological service system, the policy practice of Zhejiang Province has created a unique window for testing the interaction mechanism between the institutional environment and mental health problems (51). As a national-level pilot, Zhejiang’s experience exploration and policy response model largely reflect China’s overall direction and potential path in addressing the increasingly prominent challenges of public mental health.

Using a combination of stratified sampling and probability proportional to size (PPS) sampling, we conducted a questionnaire survey across 26 universities in 8 cities of Zhejiang Province. Strata followed the provincial higher education tiers that include Double First Class universities, provincial key universities, and other undergraduate and vocational institutions. Within each stratum we applied PPS at the university level using full time enrollment as the size measure. We computed a sampling interval, drew a random start, and selected universities by cumulative size. Data collection occurred in two stages: a pilot and a main survey. In June 2024, we distributed 97 questionnaires in a pilot test to verify the reliability and validity of the scales. The results showed that the Cronbach’s αfor the questionnaire met acceptable standards, confirming the measures’ internal consistency. The main survey was carried out from July to August 2024, yielding 680 returned questionnaires. Before case cleaning we applied prespecified inclusion–exclusion rules as part of field quality control, removing records with missing values on key fields, entries for height weight or monthly income outside predefined plausibility ranges, and very short completion times relative to the group median; on site staff also checked questionnaire completeness. After excluding invalid responses, we obtained 600 valid questionnaires for an effective response rate of 88.24%.

All survey administrators received systematic training to ensure consistency and standardization in data collection. Participants signed informed consent forms before completing the questionnaire and were informed of the study’s purpose and the anonymity of their responses. This study obtained approval from the Ethics Committee of Wenzhou Medical University, and all data were used solely for academic research.

### Measurements of variables

This study used the 5-point Likert scale with options ranging from 1 (strongly disagree) to 5 (strongly agree) to assess respondents’ attitudes to the statements. Emotional exhaustion (the mediating variable) needs a finer degree of distinction so it was measured on a seven-point Likert scale (1 = strongly disagree to 7 = strongly agree) to capture the degree of emotional depletion. Mental health (the outcome variable) was assessed with a frequency scale: respondents indicated how often they experienced certain symptoms in the past 2 weeks (“not at all,” “several days,” “more than half the days,” or “nearly every day”). Mixed response formats were retained to match the validated instruments and preserve construct sensitivity. Items followed validated instruments, and wording was harmonized for readability. Involution culture and lying flat culture were measured using validated scales developed for Chinese college samples Items for relatedness with peers, teachers and family and for survival needs were reverse coded. Higher scores indicate lower unmet need and therefore greater availability of care, support and belonging as well as greater material security. These measures capture perceived availability rather than social skill or objective income, keeping the constructs aligned with our definitions of unmet relatedness and unmet survival need. The demographic characteristics of the sample are summarized in Table 1. The measurement items, their scholarly sources, and the Chinese wording are detailed in Supplementary Tables S1, S2.

**Table 1:** Demographics of the sample.

Variables	Characteristics	Frequency	Percentage
Gender	Male	281	46.83%
	Female	319	53.17%
Age	18–22	254	42.33%
	23–26	192	32.00%
	27–30	154	25.67%
Education	Junior college (associate)	128	21.33%
	Bachelor’s degree	194	32.33%
	Master’s degree	163	27.17%
	Doctoral degree	115	19.17%
Major field	Medical sciences	103	17.17%
	Humanities & Social sciences	122	20.33%
	Arts & Sports	87	14.50%
	Engineering sciences	108	18.00%
	Natural sciences	84	14.00%
	Other	96	16.00%
Left-behind experience	Yes	232	38.67%
	No	368	61.33%
Monthly personal income (RMB)	≤2000	102	17.00%
	2000<*n* ≤ 2,500	268	44.67%
	2,500<*n* ≤ 3,000	164	27.33%
	>3,000	132	22.00%
Weekly exercise time (hour)	≤2	175	29.17%
	2<*n* ≤ 4	295	49.17%
	4<*n* ≤ 6	112	18.67%
	>6	18	3.00%

### Data analysis

Before model estimation we assessed reliability and validity by reporting coefficient alpha, conducting confirmatory factor analyses with standardized loadings, and computing composite reliability and average variance extracted; discriminant validity was examined using the Fornell-Larcker criterion. We then estimated measurement and structural models using covariance-based Structural Equation Modeling in AMOS 24.0 with maximum likelihood, evaluating model fit with *χ*^2^/df, CFI, TLI, and RMSEA, and reporting standardized path coefficients with two-tailed *p* values. To address common-method variance, beyond Harman’s single-factor test we compared a one-factor CFA against the theorized multifactor model. To complement net-effect modeling, we applied fuzzy-set Qualitative Comparative Analysis in fsQCA 3.0. Set calibration followed the direct method with anchors at the 95th percentile for full membership, the 50th percentile as the crossover, and the 5th percentile for full non-membership, aligning these cut points with the semantic high, midpoint, and low positions of the ordered response scales so that thresholds are interpretable and comparable across constructs. Truth tables were constructed with a minimum case frequency and a standard sufficiency consistency threshold, and we report intermediate and parsimonious solutions together with raw and unique coverage and overall solution consistency.

## Results

### SEM

To address common method variance at the measurement level, we compared a one-factor CFA model with the theorized multi-factor model and found a highly significant fit degradation for the one-factor solution, Δ*χ*^2^(45) = 9089.471, *p* < 0.001, indicating that a single methods factor cannot account for the covariation among indicators. Harman’s single-factor share was 33.10%, suggesting limited CMV. VIFs ranged from 1.325 to 2.030 and did not indicate problematic collinearity. The KMO was 0.937 and Bartlett’s test of sphericity was significant, confirming sampling adequacy for factor analysis. Global fit was acceptable with *χ*^2^/df = 1.877, RMSEA = 0.038, CFI = 0.968, and TLI = 0.964. Detailed item loadings, composite reliability, and average variance extracted are reported in Supplementary Tables S3. Discriminant validity follows the Fornell and Larcker criterion. The Table 2 shows the square roots of AVE and each exceeds interconstruct correlations.

**Table 2:** Discriminant validity.

Constructs	(1)	(2)	(3)	(4)	(5)	(6)	(7)	(8)	(9)	(10)
Involution culture	**0.800**									
Lying flat culture	0.374	**0.793**								
Survival need	0.463	0.317	**0.849**							
Relationship need	0.347	0.344	0.345	**0.849**						
Academic pressure	0.496	0.510	0.432	0.332	**0.808**					
Employment pressure	0.558	0.507	0.430	0.324	0.601	**0.819**				
Ruminative thinking	0.532	0.397	0.413	0.305	0.547	0.624	**0.803**			
Personal behavior	0.266	0.377	0.227	0.321	0.373	0.262	0.198	**0.809**		
Emotional exhaustion	0.483	0.408	0.425	0.271	0.507	0.533	0.433	0.592	**0.891**	
Mental health	0.241	0.323	0.249	0.272	0.270	0.325	0.263	0.260	0.248	**0.850**

Diagonal values in bold are the square roots of AVE.

**Figure 2:**
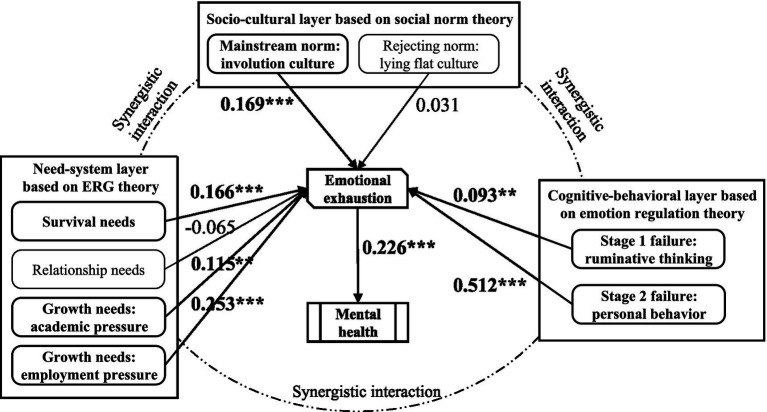
Model results. ***p<0.001, **p<0.01, *p<0.05.

The structural equation model results shown in Figure 2 indicate that personal behavior (*β* = 0.512***), ruminative thinking (*β* = 0.093*), academic pressure (*β* = 0.115**), employment pressure (*β* = 0.253***), survival need (*β* = 0.166***), and involution culture (*β* = 0.169***) have a significant positive impact on emotional exhaustion. In contrast, relationship needs (*β* = −0.065) and lying flat culture (*β* = 0.031) do not show a significant effect on emotional exhaustion. Additionally, emotional exhaustion (*β* = 0.226***) has a significant impact on mental health. To aid interpretation, we read standardized path coefficients with widely used benchmarks for effect magnitude: about 0.10 small, about 0.30 medium, at least 0.50 large, which implies a large effect for personal behavior, medium for employment pressure, and small-to-medium for survival need, academic pressure, ruminative thinking, and involution culture in this sample (Table 3).

**Table 3:** SEM path analysis result.

Hypothesis	Path	*β*	S. E.	C. R.	*p*	Result
H1	Involution culture →Emotional exhaustion	0.169	0.048	4.490	<0.001	Supported
H2	Lying flat culture →Emotional exhaustion	0.031	0.050	0.840	0.401	Not Supported
H3	Survival need →Emotional exhaustion	0.166	0.047	4.607	<0.001	Supported
H4	Relationship need →Emotional exhaustion	−0.065	0.060	−1.874	0.061	Not Supported
H5	Academic pressure →Emotional exhaustion	0.115	0.047	3.101	0.002	Supported
H6	Employment pressure →Emotional exhaustion	0.253	0.050	6.608	<0.001	Supported
H7	Ruminative thinking →Emotional exhaustion	0.093	0.051	2.509	0.012	Supported
H8	Personal behavior →Emotional exhaustion	0.512	0.077	11.188	<0.001	Supported
H10	Emotional exhaustion →Mental health	0.226	0.035	5.283	<0.001	Supported

We tested the mediating role of emotional exhaustion (H9) using bootstrapping (5,000 samples) to obtain bias-corrected confidence intervals for indirect effects (52). The bootstrap results (see Table 4) showed that emotional exhaustion significantly mediated the effect of each independent variable on mental health. For all eight predictors—personal behavior, rumination, academic pressure, employment pressure, relationship need, survival need, involution culture, and lying flat culture—the indirect effect via emotional exhaustion was significant (the 95% confidence intervals for indirect effects did not include zero). Thus, H9 is supported, indicating that emotional exhaustion serves as a significant mediator between each of the multi-level stressors and mental health outcomes.

**Table 4:** Results of bootstrap mediation effect test.

Latent variable	Effect	Value	95% CI		Relative mediating effect	Result
			LLCI	ULCI		
Involution culture	Total effect	0.213	0.135	0.291	35.95%	Supported
	Direct effect	0.137	0.051	0.222		
	Indirect effect	0.077	0.037	0.117		
Lying flat culture	Total effect	0.288	0.211	0.365	19.64%	Supported
	Direct effect	0.231	0.149	0.313		
	Indirect effect	0.057	0.024	0.089		
Survival need	Total effect	0.221	0.140	0.302	34.98%	Supported
	Direct effect	0.144	0.056	0.232		
	Indirect effect	0.077	0.037	0.121		
Relationship need	Total effect	0.299	0.217	0.381	17.46%	Supported
	Direct effect	0.247	0.163	0.331		
	Indirect effect	0.052	0.027	0.081		
Academic pressure	Total effect	0.236	0.160	0.312	30.40%	Supported
	Direct effect	0.164	0.080	0.249		
	Indirect effect	0.072	0.028	0.114		
Employment pressure	Total effect	0.282	0.208	0.355	20.60%	Supported
	Direct effect	0.224	0.140	0.307		
	Indirect effect	0.058	0.014	0.103		
Ruminative thinking	Total effect	0.234	0.157	0.311	28.37%	Supported
	Direct effect	0.167	0.085	0.250		
	Indirect effect	0.066	0.031	0.102		
Personal behavior	Total effect	0.205	0.137	0.272	36.20%	Supported
	Direct effect	0.131	0.050	0.211		
	Indirect effect	0.074	0.031	0.116		

### fsQCA

Each variable was calibrated using the direct method based on theoretical knowledge and data distribution: we set the threshold for full membership at the 95th percentile, the crossover point at the 50th percentile, and full non-membership at the 5th percentile (53). Through calibration, raw variable scores were transformed into fuzzy membership values ranging from 0 to 1 (Table 5 provides descriptive statistics and calibration anchors for each condition). Necessity tests showed no single condition exceeded the 0.90 consistency threshold, so there is no single necessary factor for either high risk or low risk outcomes. Necessity details are moved to Supplementary Table S4 (54).

**Table 5:** Descriptive statistics.

Variable	Mean	Std. dev.	Full membership (95%)	Cross over point (50%)	Full non-membership (5%)
Involution culture	2.671	1.035	4.333	2.667	1.000
Lying flat culture	2.707	1.021	4.333	2.667	1.000
Survival need	2.503	0.993	4.333	2.333	1.000
Relationship need	2.638	0.964	4.333	2.667	1.000
Academic pressure	2.733	1.051	4.333	2.667	1.000
Employment pressure	2.685	1.070	4.333	2.667	1.000
Ruminative thinking	2.773	1.036	4.333	2.667	1.000
Personal behavior	2.931	1.183	4.667	2.667	1.333
Emotional exhaustion	3.206	1.564	6.143	2.857	1.143
Mental health	2.124	1.025	3.857	1.714	1.000

Next, we conducted a sufficiency analysis to identify combinations of conditions linked to the outcome of poor mental health. Given that our dataset is relatively large (*N* > 150), we set the minimum case frequency threshold to 3, the minimum consistency threshold to 0.80, and the proportional reduction in inconsistency (PRI) threshold to 0.70 for truth table analysis (21). We obtained complex, parsimonious, and intermediate solutions. Following standard practice, the intermediate solution is reported and core versus peripheral conditions are distinguished algebraically: a condition is core if it appears in both the parsimonious and the intermediate solutions, and peripheral if it appears only in the intermediate solution under directional expectations. The results of the sufficiency analysis are shown in Table 6.

**Table 6:** Truth table.

Conditional configuration	S1a	S1b	S2	S3a	S3b
Involution culture	●	●	●	●	●
Lying flat culture		•	•	●	●
Survival need	→		●	●	●
Relationship need	●	●	→	●	●
Academic pressure	⊗	⊗	•	●	●
Employment pressure	●	●	•	●	●
Ruminative thinking	●	●	•	⊗	●
Personal behavior	●	●	●	●	⊗
Emotional exhaustion	•	•	•	●	●
raw coverage	0.222	0.251	0.259	0.248	0.243
unique coverage	0.009	0.012	0.027	0.025	0.037
consistency	0.916	0.899	0.908	0.903	0.913
solution coverage	0.385				
solution consistency	0.866				

● or • indicates the presence of a condition; ⊗ or → indicates the absence of a condition; ● or ⊗ indicates a core condition; • or → indicates a peripheral condition. Spaces indicates that a condition may or may not be present.

According to the configurational results presented in Table 6, a total of five pathways drive high mental health risk. The overall consistency of 0.866 indicates that 86.6% of the samples exhibit high mental health risk outcomes, and each pathway has a consistency greater than 0.8 (54), suggesting that these five pathways serve as sufficient conditions for high mental health risk. The total coverage of the configurational model is 0.386, meaning that these pathways explain 38.6% of the cases. These five pathways can be classified into three types.

#### Configuration A

This family covers roughly one quarter of the high-risk cases with high internal consistency. The configuration is anchored by involution culture, employment pressure, unmet relationship need, ruminative thinking, and maladaptive personal behavior as core conditions. Academic pressure does not enter as a core condition and lying flat culture is not required. The pattern indicates that high competition together with thin relational resources and regulation shortfalls consistently co-occurs among a sizable subset of high-risk students.

#### Configuration B

This configuration accounts for the largest share of high-risk cases and exhibits high consistency. It is anchored by unmet survival need, involution culture, and maladaptive personal behavior as core conditions, with academic pressure, employment pressure, rumination, and emotional exhaustion entering peripherally, and relationship need not required. The pattern indicates that material strain combined with competition-oriented norms is a common signature among high-risk cases.

#### Configuration C

The two variants each account for about one quarter of high-risk cases and both show high consistency. Shared core conditions are involution culture, lying flat culture, academic pressure, employment pressure, and emotional exhaustion. One variant closes through maladaptive personal behavior, the other through rumination. The family indicates that high demands under mixed cultural signals co-occur with either cognitive or behavioral regulation shortfalls among a substantial portion of high-risk cases.

The results indicate that personal behavior, employment pressure, involution culture, and emotional exhaustion are more critical factors contributing to high mental health risk compared to other antecedent conditions.

In the robustness test, referring to previous literature, the PRI consistency threshold was adjusted to 75%, with the completely membership point and completely non-membership point set to 90 and 10%, respectively, while the crossover point remained at 50% for two types of tests (55). The test results show that the model configuration changed minimally and exhibited a clear subset relationship, indicating that the results are robust.

## Discussion and conclusions

### Discussion

This study examined college student mental health through a social ecological lens and verified that emotional exhaustion mediates the effects of multi-level variables on mental health using SEM, while fsQCA uncovered conjunctural pathways that cannot be reduced to average effects. By integrating symmetric evidence from SEM with asymmetric evidence from fsQCA, we move from single predictor logic to configuration logic. This triangulation aligns with the social ecological view that individuals are embedded in layered contexts. Across the model, emotional exhaustion emerges as a transmission hub through which macro pressures, meso constraints and micro dysregulation reach mental health.

At the macro level, SEM shows that involution culture carries a positive association with emotional exhaustion through a pathway consistent with effort reward imbalance (56). This pathway is not only statistical but also motivational since perceived unfairness invites continued overcommitment that drains regulatory resources, a pattern repeatedly observed in the effort reward tradition. fsQCA sharpens this picture by showing that involution is a core component across configurations, indicating that cultural and institutional pressure becomes decisive when it combines with meso and micro stressors. In configuration A involution travels with high employment pressure and rumination, which turns social comparison into a persistent appraisal of uncontrollable demand. In configuration B involution fuses with survival anxiety and maladaptive behavior, signalling cultural internalization of effort determinism under scarcity. The non-significant average effect of lying flat in SEM is therefore not a paradox but a sign of functional heterogeneity. In Chinese student samples lying flat often operates as a situational tactic rather than a stabilized value in the cultural toolkit sense, which weakens any uniform net association with exhaustion (57). fsQCA clarifies that in configuration C lying flat coexists with high academic and employment pressure where the tactic becomes disengaged avoidance and joins the overload mechanism. Cross cultural evidence explains why the same tactic can appear adaptive or harmful. Western recovery research distinguishes psychological detachment that restores resources during off time from disengagement that undermines recovery when stressors remain high and documents a recovery paradox under heavy stress (58). This suggests a threshold account that fits our mixed evidence. When demands pass a certain level the short term benefits of disengaging are overshadowed by resource loss spirals, so the tactic is non protective on average yet central inside a high strain constellation. SEM captures small average effects while fsQCA reveals conjunctural sufficiency. Structural competition is the macro driver and the meaning of lying flat is contingent on the stress ecology it inhabits (59).

At the meso layer survival pressure centered on economic precarity shows a robust direct path to exhaustion in SEM and emerges as a core ingredient of configuration B. This aligns with meta analytic evidence that job insecurity and financial strain predict poorer mental health and with reports that uncertainty about future prospects amplifies cognitive load (60). fsQCA adds that when survival anxiety couples with cultural internalization and maladaptive behavior the configuration is sufficient for poor outcomes, which fits a loss spiral account in conservation of resources theory (61). Academic and employment pressures form a dual axis that undermines perceived control and induces future focused load. In configuration A high employment pressure partners with macro competition and rumination and the trio maps onto a rigidity pattern that pushes students toward narrowing goals and avoidance. By contrast the path from unmet relationship need to exhaustion is not significant in SEM. This is consistent with the instrument content and the cultural context. The scale uses reverse scoring across family teacher and peer domains so higher scores reflect lower unmet need and the items index relationship management ability more than receipt of explicit aid. In East Asian contexts such competence aligns with a preference for implicit support that works without overt disclosure so the direct link to exhaustion is diluted at the average level (62). Western buffering models emphasize explicit support while cross cultural studies show that Asians and Asian Americans often gain more from implicit affiliation than from explicit help seeking which can itself be stressful under harmony norms (63). fsQCA is sensitive to such thresholds and detects relational factors as contributory inside configuration A when social comparison and self-regulation failures are also present. This integrated reading reconciles the small symmetric path with the configurational salience and clarifies that relationship processes protect only when matched to the stressor profile and timing.

At the micro layer cognitive and behavioral regulation failures drive exhaustion. Rumination narrows attention, prolongs negative affect, and impairs instrumental action, and our SEM identifies a positive path from rumination to exhaustion (64). Configuration A shows rumination as a core ingredient in the presence of macro competition and employment pressure which indicates that repetitive negative thinking becomes the final common pathway once contextual demands saturate capacity. Maladaptive behaviors such as curtailed sleep or stimulant overuse may blunt distress transiently yet accumulate cost and escalate allostatic load (65). Configuration B underscores this route because survival anxiety and cultural internalization push students into self exploitation that trades short term relief for long term strain. Evidence from university settings shows that improving sleep quality produces reliable mental health gains and reduces rumination and that digital and group delivered programs can achieve moderate effects at scale (66–68). Configuration C illustrates system overload where either rumination or maladaptive behavior can carry the overload forward after macro and meso stressors have combined, which explains why average effects in SEM remain modest while sufficiency appears in fsQCA. This pattern highlights a practical point that micro level habits are modifiable levers that transmit higher level pressures and that targeting these levers can shift outcomes even when upstream conditions change slowly.

### Conclusion

By integrating SEM and QCA, our study concludes that emotional exhaustion among contemporary Chinese college students is not just an individual psychological problem, but has become a systemic issue. This problem is shaped by macro-level cultural and institutional pressures, and multiple stressors permeate into the domain of mental health through social-cognitive processes. The above findings provide a deeper multi-level understanding of college student mental health problems and offer theoretical support for designing subsequent intervention strategies.

### Social implications

Primary prevention aims at the whole student population and is led by national and provincial education authorities together with university leadership. Actions include embedding mental health into medium and long term education and employment planning and implementing campus wide literacy and screening at matriculation and mid-term, aligned with recent national action plans. Universities operationalize this through general education modules on mental health and recovery skills and through population level sleep health programs and structured peer networks (69). Secondary prevention targets identifiable at risk groups and is executed by university counseling centers academic affairs offices and career services using stepped care pathways that link low intensity digital or group programs to rapid escalation when indicators rise and that add targeted supports for students facing financial strain or job insecurity (70). Tertiary prevention focuses on students with established disorders or crisis risk and is delivered through coordinated referral agreements between counseling centers and designated hospitals with case management and return to study plans led by counselors and student affairs units. Monitoring relies on simple audit metrics such as coverage rates time to first contact and symptom change at follow up which makes the program accountable to both administrators and students (71).

### Academic implications

This study, by constructing a research framework from a social ecological perspective, transcends the limitations of traditional single-level mental health research and provides a systematic analytical tool for exploring the impact mechanism of emotional exhaustion on mental health. Unlike prior studies that focused on a single stressor or individual trait (72), our research confirms from a more macro perspective the social embeddedness of mental health problems in college students (73). By demonstrating the mediating role of emotional exhaustion between various predictors and mental health, we extend the theoretical scope of emotional exhaustion: we reconceptualize it from merely an individual stress response to a social-ecological transmission mechanism, thereby broadening the explanatory boundaries of traditional frameworks (74). Additionally, the use of an integrated SEM and fsQCA approach verifies both linear causal pathways and asymmetric configurational effects among variables, providing a methodological innovation for the field (75). These advances push the paradigm of mental health research from individual-centric attribution toward a socio-ecological co-evolutionary perspective, laying the groundwork for future development of multi-dimensional dynamic models and culturally contextualized research.

## Limitations and future lines of research

This study has four main limitations. First, the cross-sectional design prevents us from capturing the temporal dynamics of emotional exhaustion, highlighting the need for longitudinal tracking to clarify stress transmission over time. Second, the sample is limited to Zhejiang Province, which may constrain generalizability. Moreover, culturally specific constructs such as involution and lying flat culture may not extend beyond China, requiring cross-cultural comparative studies to test their applicability. Third, reliance on self-reported questionnaires introduces potential biases, including social desirability and common method variance, suggesting future research should incorporate multi-source and multimodal data. Fourth, although macro–meso–micro levels were described as relatively independent, our fsQCA analysis has already partly revealed cross-level configurations. This preliminary contribution should be explicitly acknowledged and further expanded to explore the nonlinear coupling effects between cultural-cognitive and institutional pressures. Future research should therefore build multi-wave longitudinal databases, conduct cross-cultural comparisons, and integrate physiological indicators with socio-ecological variables. These steps would deepen our understanding of how structural and cultural pressures interact with resource dynamics to shape psychological resilience.

## Data Availability

The original contributions presented in the study are included in the article/Supplementary material, further inquiries can be directed to the corresponding authors.
