# Supplementary material for: How does emotional exhaustion among Chinese college students affect mental health? A mixed-methods study in Zhejiang, China
Source: Front Public Health. 2025 Oct 28;13:1669092. doi: 10.3389/fpubh.2025.1669092 (PMC12602391; doi:10.3389/fpubh.2025.1669092)
Supplement: Supplementary file 2 [file Supplementary_file_1.docx]

**Supplementary Materials**

This supplementary file provides measurement materials and validation summaries. S1 lists the English questionnaire items with scholarly sources. S2 presents the finalized Chinese items after forward and back translation. S3 reports composite reliability and convergent validity for all latent constructs. S4 presents the analysis of necessary conditions for the outcome and reports consistency and coverage for both presence and absence of conditions.

**Table S1 Measuring scales**

| **Construct** | **Items** | **Reference** |
| --- | --- | --- |
| Involution culture | Even after completing a task, I still put in additional effort to perfect it. | (1) |
|  | I have a strong atmosphere of competition with my classmates. |  |
|  | Seeing others outperform me, I do my utmost to emulate and catch up. |  |
| Lying-flat culture | Grades in school don’t matter much; just passing is sufficient. | (2) |
|  | I don’t want to participate in any social activities. |  |
|  | I find no meaning in life, so I simply do nothing. |  |
| Survival needs (unmet) | My family’s financial situation is better than that of most of my peers. | (3) |
|  | The living allowance from my parents (or my own income) is enough to cover my expenses. |  |
|  | I do not habitually borrow money or incur debts. |  |
| Relationship needs (unmet) | I can handle relationships with my peers very well. | (4) |
|  | I maintain good relationships with my teachers (including advisors). |  |
|  | I handle relationships with my family members (e.g., parents) well. |  |
| Academic pressure | I find my major courses difficult, and some are very strenuous for me. | (5) |
|  | Having too many study tasks leaves me not knowing where to start. |  |
|  | I feel I have no motivation to study and regret wasting time. |  |
| Employment pressure | I am unsure what kind of job I want in the future and lack specialized skills. | (6) |
|  | I feel worried and very uncertain about the job prospects in my field of study. |  |
|  | I feel my competitiveness in the job market is not as good as that of others. |  |
| Ruminative thinking | I repeatedly think about past stressful or upsetting events and find it hard to stop. | (7) |
|  | I keep analyzing why something happened rather than how to solve the problem. |  |
|  | I continually dwell on bad experiences and feel regretful. |  |
| Personal behavior (maladaptive) | When feeling upset, I might choose to smoke to relieve my emotions. | (8) |
|  | When feeling depressed, I might choose to drink alcohol to feel better. |  |
|  | When feeling down, I might choose to stay up late to cope with my feelings. |  |
| Emotional exhaustion | Even if I haven’t done much, I feel completely exhausted. | (9) |
|  | I often postpone or avoid tasks out of fear of failure. |  |
|  | Seeing others do better than me makes me feel intense anxiety or self-doubt. |  |
|  | I set very high standards for myself—no matter how well I do, I still feel it isn’t good enough. |  |
|  | I find it hard to feel enjoyment or motivation from daily activities. |  |
|  | I often feel incapable of handling life’s challenges. |  |
|  | Small things easily cause my emotions to fluctuate or even collapse. |  |
| Mental health | Trouble concentrating to the extent it affects study or work. | (10) |
|  | Feeling down or depressed. |  |
|  | Difficulty falling asleep, staying asleep, or sleeping too much. |  |
|  | Thoughts that you would be better off dead or of hurting yourself in some way. |  |
|  | Poor appetite or overeating, with noticeable weight change. |  |
|  | Feeling bad about yourself—thinking you are a failure or have let yourself or your family down. |  |
|  | Feeling fidgety. |  |

**Table S2 Chinese Questionnaire Items**

| 构念 | 条目 |
| --- | --- |
| 内卷文化 | 即便已经完成了任务，我还是会做出更多努力去进行完善 |
|  | 我与同学间有强烈的竞争氛围 |
|  | 看到别人比自己优秀，我会拼尽全力效仿与追赶 |
| 躺平文化 | 学校里的成绩没有太大意义，及格就行 |
|  | 我不想参加任何社交活动 |
|  | 我找不到人生的意义，所以干脆什么都不做 |
| 生存需要 | 我觉得我家的经济状况在同龄人之中比较好 |
|  | 父母给的生活费（或个人收入）足够支撑我的日常开销 |
|  | 我没有经常借钱和欠款的习惯 |
| 关系需要 | 我能很好处理和同辈的关系 |
|  | 我能很好处理和老师（包括导师）的关系 |
|  | 我能很好处理和家庭成员（父母等）的关系 |
| 学业压力 | 我觉得专业课难，有些专业课程的学习令我感到很吃力 |
|  | 学习任务过多让我不知道从何入手 |
|  | 我觉得我没有学习动力，因浪费时间而懊悔 |
| 就业压力 | 我不知道未来希望从事什么样的工作，没有一技之长 |
|  | 我对我所学专业就业前景感到担忧，十分迷茫 |
|  | 我觉得我的职场竞争力不如别人 |
| 反刍思维 | 我反复回想过去有压力或难过的事情，难以停止 |
|  | 我会不断分析“为什么会发生这种事”，而不是如何解决 |
|  | 我总在回忆曾经那些不好的事，总在后悔 |
| 个人行为 | 沮丧时我可能会选择抽烟来缓解情绪 |
|  | 沮丧时我可能会选择饮酒来缓解情绪 |
|  | 沮丧时我可能会选择熬夜来缓解情绪 |
| 情绪耗竭 | 即使没有做太多事情，我也感到精疲力尽 |
|  | 我经常因为害怕失败而推迟或逃避需要完成的任务 |
|  | 当看到别人比我优秀时，我会感到强烈的焦虑或自我怀疑 |
|  | 我对自己要求非常严格，即使事情做得不错，仍觉得不够完美 |
|  | 我很难从日常活动中感受到乐趣或动力 |
|  | 我经常觉得自己没有能力应对生活中的挑战 |
|  | 我容易因为小事而情绪波动，甚至感到崩溃 |
| 心理健康 | 我发现自己难以集中注意力，甚至影响学习或工作 |
|  | 感到心情沮丧或低落 |
|  | 入睡困难、睡不安稳或睡眠过多 |
|  | 有不如用死掉或者某种方式伤害自己的念头 |
|  | 食欲不振或吃太多，体重发生显著变化 |
|  | 觉得自己很糟、很失败、让自己或家人失望 |
|  | 烦躁，坐立不安 |

**Table S3 Composite reliability and convergent validity**

| **Variables** | **Standardised factor loading** | **AVE** | **CR** | **Cronbach’s alpha** |
| --- | --- | --- | --- | --- |
| Involution culture | 0.797 - 0.803 | 0.641 | 0.842 | 0.842 |
| Lying-flat culture | 0.782 - 0.804 | 0.629 | 0.836 | 0.835 |
| Survival need | 0.768 - 0.935 | 0.720 | 0.885 | 0.871 |
| Relationship need | 0.682 - 0.965 | 0.721 | 0.884 | 0.872 |
| Academic pressure | 0.799 - 0.826 | 0.653 | 0.85 | 0.849 |
| Employment pressure | 0.799 - 0.830 | 0.670 | 0.859 | 0.858 |
| Ruminative thinking | 0.780 - 0.815 | 0.645 | 0.845 | 0.845 |
| Personal behavior | 0.668 - 0.888 | 0.655 | 0.849 | 0.837 |
| Emotional exhaustion | 0.846 - 0.909 | 0.793 | 0.964 | 0.964 |
| Mental health | 0.842 - 0.866 | 0.722 | 0.948 | 0.949 |

**Table S4 Analysis of the necessary conditions**

| **Conditions tested** | **Outcome variable:**  **Mental health risk** | | **Outcome variable:**  **~Mental health risk** | |
| --- | --- | --- | --- | --- |
|  | Consistency | Coverage | Consistency | Coverage |
| Involution culture | 0.688 | 0.667 | 0.561 | 0.578 |
| ~Involution culture | 0.564 | 0.548 | 0.677 | 0.698 |
| Lying-flat culture | 0.710 | 0.675 | 0.564 | 0.570 |
| ~Lying-flat culture | 0.548 | 0.542 | 0.678 | 0.713 |
| Survival need | 0.694 | 0.686 | 0.548 | 0.575 |
| ~Survival need | 0.522 | 0.515 | 0.700 | 0.708 |
| Relationship need | 0.684 | 0.684 | 0.551 | 0.585 |
| ~Relationship need | 0.585 | 0.551 | 0.703 | 0.702 |
| Academic pressure | 0.710 | 0.659 | 0.569 | 0.560 |
| ~Academic pressure | 0.525 | 0.534 | 0.653 | 0.705 |
| Employment pressure | 0.721 | 0.689 | 0.536 | 0.543 |
| ~Employment pressure | 0.522 | 0.515 | 0.693 | 0.725 |
| Ruminative thinking | 0.725 | 0.658 | 0.585 | 0.564 |
| ~Ruminative thinking | 0.520 | 0.542 | 0.645 | 0.713 |
| Personal behavior | 0.707 | 0.643 | 0.585 | 0.565 |
| ~Personal behavior | 0.522 | 0.542 | 0.631 | 0.696 |
| Emotional exhaustion | 0.692 | 0.690 | 0.533 | 0.564 |
| ~Emotional exhaustion | 0.562 | 0.531 | 0.707 | 0.709 |

**Reference**

1. Yan D, Zhang H, Guo S, Zeng W. Influence of anxiety on university students' academic involution behavior during COVID-19 pandemic: Mediating effect of cognitive closure needs. *Frontiers in psychology*. (2022)13:1005708–. doi: 10.3389/fpsyg.2022.1005708

2. Liu A, Shi Y, Zhao Y, Ni J. Influence of academic involution atmosphere on college students' stress response: the chain mediating effect of relative deprivation and academic involution. *Bmc Public Health*. (2024)24(1). doi: 10.1186/s12889-024-18347-7

3. Deckard FM, Goosby BJ, Cheadle JE. Debt Stress, College Stress: Implications for Black and Latinx Students' Mental Health. *Race and social problems*. (2022)14(3):238–53. doi: 10.1007/s12552-021-09346-z

4. Xu Q, Li S, Yang L. Perceived social support and mental health for college students in mainland China: the mediating effects of self-concept. *Psychology Health & Medicine*. (2019)24(5):595–604. doi: 10.1080/13548506.2018.1549744

5. Chen Y. The relationship between academic stress and mental health: Resilience as a moderator. *Social Behavior and Personality: an international journal*. (2024)52(12):1–9. doi:

6. Peng Y, Lv SB, Low SR, Bono SA. The impact of employment stress on college students: psychological well-being during COVID-19 pandemic in China. *Current psychology (New Brunswick, NJ)*. (2023):1–12. doi: 10.1007/s12144-023-04785-w

7. Treynor W, Gonzalez R, Nolen-Hoeksema S. Rumination reconsidered: A psychometric analysis. *Cognitive therapy and research*. (2003)27:247–59. doi:

8. Caamano-Navarrete F, Saavedra-Vallejos E, Guzman-Guzman IP, Arriagada-Hernandez C, Fuentes-Vilugron G, Jara-Tomckowiack L, et al. Unhealthy Lifestyle Contributes to Negative Mental Health and Poor Quality of Life in Young University Students. *Healthcare*. (2024)12(22). doi: 10.3390/healthcare12222213

9. Dyrbye LN, Thomas MR, Massie FS, Power DV, Eacker A, Harper W, et al. Burnout and suicidal ideation among US medical students. *Annals of Internal Medicine*. (2008)149(5):334–W70. doi: 10.7326/0003-4819-149-5-200809020-00008

10. Kroenke K, Spitzer RL, Williams JBW. The PHQ-9 - Validity of a brief depression severity measure. *Journal of General Internal Medicine*. (2001)16(9):606–13. doi: 10.1046/j.1525-1497.2001.016009606.x
